# Correction to: Excitable networks for finite state computation with continuous time recurrent neural networks

**DOI:** 10.1007/s00422-021-00911-8

**Published:** 2021-11-18

**Authors:** Peter Ashwin, Claire Postlethwaite

**Affiliations:** 1 Center for Systems, Dynamics and Control, Department of Mathematics, University of Exeter, Exeter, EX4 4QF UK; 2 Department of Mathematics, University of Auckland, Auckland, 1142 New Zealand

## Correction to: Biological Cybernetics (2021) 115:519–538 10.1007/s00422-021-00895-5

The Excitable networks for finite state computation with continuous time recurrent neural networks, written by Peter Ashwin and Claire Postlethwaite, was originally published electronically in SpringerLink on 05 October 2021 without open access. After publication volume 115, issue 5, pages 519–538 With the author(s)’ decision to opt for Open Choice the copyright of the article changed on 6 November 2021 to © The Author(s) 2021 and the article is licensed under a Creative Commons Attribution 4.0 International License, which permits use, sharing, adaptation, distribution and reproduction in any medium or format, as long as you give appropriate credit to the original author(s) and the source, provide a link to the Creative Commons licence, and indicate if changes were made. The images or other third party material in this article are included in the article’s Creative Commons licence, unless indicated otherwise in a credit line to the material. If material is not included in the article’s Creative Commons licence and your intended use is not permitted by statutory regulation or exceeds the permitted use, you will need to obtain permission directly from the copyright holder. To view a copy of this licence, visit http://creativecommons.org/licenses/by/4.0/.

The original article has been corrected.

